# Evaluation of p(HEMA-co-NIPAM) hydrogels for removal of methylene blue from aqueous solution: isotherm, kinetic, and thermodynamic studies

**DOI:** 10.55730/1300-0527.3662

**Published:** 2024-01-22

**Authors:** Hayriye Mine ANTEP, Münire Nalan DEMİR, Cevher GÜNDOĞDU HIZLIATEŞ, Simge ÖZTÜRK, Elif ESEN COŞKUN

**Affiliations:** 1 Department of Chemistry, Dokuz Eylül University, İzmir, Turkiye; 2 Graduate School of Natural and Applied Science, Dokuz Eylül University, İzmir, Turkiye; 3 Vestel Trade Corporation, Manisa, Turkiye

**Keywords:** Adsorption, methylene blue, hydrogel, kinetics, adsorption isotherms

## Abstract

In this study, a novel adsorbent material, poly (2-hydroxyethyl methacrylate-co-N-isopropyl acrylamide) (p(HEMA-co-NIPAM) hydrogel, was synthesized for the purpose of removing methylene blue (MB) from aqueous media. The synthesis of hydrogel was carefully conducted, and its properties were thoroughly examined using techniques such as Fourier-transform infrared spectroscopy (FTIR), scanning electron microscopy (SEM), and thermogravimetric analysis (TGA). The adsorption experiments conducted revealed a remarkable affinity of p(HEMA-co-NIPAM) hydrogel towards MB. The highest adsorption was observed when 0.05 g of the adsorbent were utilized, with optimal conditions at a pH of 6.0 and a temperature of 15 °C. This underscores the importance of pH control and temperature regulation in optimizing the adsorption treatment. The adsorption behavior of MB on p(HEMA-co-NIPAM) hydrogel was best elucidated by the Langmuir isotherm model, which provided insights into the maximum adsorption capacity. Impressively, this capacity reached 126.6 mg/g, indicative of the adsorbent’s robust capability to capture the MB molecules. The isotherm data further highlighted the strong affinity between p(HEMA-co-NIPAM) hydrogel and the MB dye, underscoring the effectiveness of the synthesized hydrogel as an adsorbent material. The successful application of p(HEMA-co-NIPAM) hydrogel for MB adsorption not only emphasizes its potential for wastewater treatment but also hints at its broader significance for environmental remediation. By harnessing the adsorption capabilities of this hydrogel, the removal of MB from industrial and domestic wastewater could be significantly enhanced, leading to cleaner water resources. This study presents p(HEMA-co-NIPAM) hydrogel as a promising adsorbent material with exceptional affinity for MB. This is demonstrated through a comprehensive analysis of its synthesis, characterization, and adsorption performance. The findings hold promise for addressing wastewater contamination issues and promoting sustainable water management practices.

## 1. Introduction

It is projected that the world population will surpass 10 billion by the year 2050, leading to an urgent escalation in the demand for clean water and secure food production. Given that water is a paramount resource for human survival, the surge in industrial wastewater discharge into water bodies has magnified the significance of water pollution worldwide. Among the various categories of wastewater, particular attention is being directed towards dye-contaminated wastewater, primarily due to the continuous advancement of printing and dyeing industrial processes. The spectrum of industrial sectors, including textiles, leather, paper, rubber, printing, and plastics, employs over 10,000 distinct dyes and pigments. This industrialization results in an annual global synthesis of nearly 0.7 million tons of dyes [1]. This increasing use of dyes has raised concerns due to the intrinsic properties of certain types, including acidic, basic, azo, diazo, anthraquinone, disperse-based, and metal complex variations [2,3]. Many of these dyes, particularly those derived from benzidine and naphthalene, exhibit carcinogenic and mutagenic attributes that pose risks to human, animal, and aquatic organisms. Exposure to these dyes has been associated with negative health effects, such as harm to kidneys, liver, brain, reproductive systems, and the central nervous system, as well as skin irritation [1,4]. The illegal discharge of wastewater compounds these challenges, precipitating severe environmental contamination. To address the deleterious impact of dye-contaminated wastewater on human health and the environment, it is crucial to pretreat the wastewater before releasing it into the main waterways. Comprehensive wastewater treatment can mitigate the detrimental consequences of dye-contaminated wastewater, thereby alleviating potential harm to both humans and the ecosystem. As the global population continues to grow, the effective management of dye-related pollution will play an integral role in safeguarding water resources and ensuring the sustenance of a healthier and more sustainable future [5].

Over the past few years, a variety of techniques encompassing physical, chemical, and biological methodologies have been developed to address the removal of dyes wherefrom wastewater. Notable methods include coagulation and flocculation [6], ozonation [7], electrochemical processes [8], fungal decolorization [9], and adsorption [10,11]. Among these techniques, adsorption is one of the most commonly used procedures for dye removal. Its popularity can be attributed to its simplicity, cost-effectiveness, and remarkable efficiency [1,12]. Activated carbon has emerged as a preeminent adsorbent, finding extensive use in this domain. However, over the past decades, a multitude of innovative adsorbents has been developed and customized for the removal of dyes. These include a range of materials from clays and biosorbents to industrial byproducts and various other substances [13–15]. Notably, functional polymeric adsorbents, such as hydrogels, have gained great attention due to their exceptional attributes. These attributes encompass elevated selectivity, regenerative potential, structural diversity, robust physicochemical stability, and an environmentally friendly nature [16]. Hydrogels, in particular, have emerged as a notable category of functional polymeric adsorbents. Their appeal lies in their capacity for selective adsorption, coupled with the ability for regeneration, ensuring sustained efficacy over time. Furthermore, the versatile structural configurations of hydrogels reinforce tailored design to match specific adsorption requirements. Their robust physicochemical stability ensures a prolonged operational lifespan, while their eco-friendly disposition aligns with sustainable practices. Considering these advantages, the utilization of functional polymeric adsorbents such as hydrogels offers a promising avenue for effective and sustainable dye removal. As the field continues to evolve, these advancements contribute to refining our capability to combat the challenges posed by dye-contaminated wastewater and underscore the crucial role of innovation in environmental remediation [16].

Methylene blue (MB) is an aromatic heterocyclic chemical compound characterized by its thiazine structure, with a molecular formula of C_16_H_18_N_3_SCl. With cationic groups, MB finds extensive application in industries such as textiles, printing, and leather. Additionally, it is a constituent of both human and veterinary pharmacopoeias. Despite its widespread use, MB poses significant threats due to its toxic, carcinogenic, and nonbiodegradable nature, which presents potential hazards to both human health and the environment. The properties of the compound raise concern due to its toxicity and carcinogenicity, which pose risks to human health. Furthermore, its inability to biodegrade increases the risk of long-term environmental damage. Industries that rely on MB as a resource must balance its functional utility with the need for safeguarding human and ecological health. Exposure to MB poses a range of health hazards for humans. Individuals subjected to this compound may experience a spectrum of health risks, including nausea, respiratory distress, abdominal disorders, visual impairment, gastrointestinal and mental disturbances, cyanosis, methemoglobinemia, tissue necrosis, shock, and jaundice [17,18]. These potential health outcomes emphasize the urgency of addressing MB contamination in various settings. The broader implications of MB’s toxicity underscore the necessity of adopting strategies that minimize its usage, promote safer alternatives, and prioritize effective removal techniques from wastewater. A holistic approach is imperative to mitigate the negative potency of MB on human health and the environment, while still addressing to the demands of industries that rely on this compound.

The synthesis and characterization of hydrogels featuring a range of functional groups have gained significant global attention as a research frontier. This exploration aims to improve the adsorption efficiency of adsorbents. Consequently, the application of hydrogels in wastewater treatment has garnered attention due to their remarkable capacity for adsorption, recoverability, and reusability [16]. The attributes of hydrogels, particularly their preeminent adsorption capacity and potential for recovery, contribute to their appeal as a promising solution for addressing wastewater challenges. For instance, poly(2-hydroxyethyl methacrylate) (pHEMA) has emerged as a hydrogel of interest, owing to its exceptional biocompatibility, chemical stability, ease of preparation, and ability to adopt diverse shapes [19]. The versatility of pHEMA as a hydrogel is further enhanced when it is combined and polymerized with other chains, such as methacrylic acid, polyvinyl alcohol, or maleic anhydride (MAH), which enhances its adsorption capacity after functionalization [20]. Hernandez-Martínez et al. investigated the potential of a copolymer of 2-hydroxyethyl methacrylate and N,N-dimethylacrylamide (p(HEMA-co-DMAa)) as an effective adsorptive matrix in their research [21]. The incorporation thermo-sensitive polymers into hydrogels has also shown potential. As stated in the article by Naseem et al. [22], stimuli-responsive adsorbents, such as poly(N-isopropylacrylamide) (PNIPAM) based hydrogels, have significant advantages compared to other adsorbent materials. Due to their crosslinked polymeric structure, these materials exhibit reversible transitions in swelling or deswelling in response to various stimuli, including changes in pH, temperature, and ionic strength. As known, temperature-responsive hydrogels such as poly(N-isopropylacrylamide) (PNIPAM) demonstrate a specific volume phase transition temperature (VPTT). Below this VPTT, the hydrophilic amide groups within these hydrogel particles form hydrogen bonds with water molecules, resulting in a notable increase in the adsorption of hydrophilic dyes. Conversely, above the VPTT, hydrogen bonding is either weakened or disrupted. The adsorption of hydrophobic dyes increases due to the heightened hydrophobicity of the polymer at high temperatures. Consequently, the hydrophilic/hydrophobic properties of hydrogel particles adjust their ability to remove dyes from aqueous solutions based on their inherent characteristics. Moreover, the adsorption capacity of hydrogel particles for dye molecules can be precisely adjusted as needed by using various monomers. The copolymerization of hydrophilic monomers, such as HEMA, with NIPAM shifts the VPTT of NIPAM-based hydrogel particles to higher temperatures [22]. Combining NIPAM with specific functional polymers or monomers has also yielded versatile polymeric structures applicable across various fields such as chromatography, agriculture, food industry, affinity precipitation, and controlled biocatalysis. Jain et al. employed the cryogelation technique to synthesize poly(2-hydroxyethyl methacrylate-co-N-isopropyl acrylamide) (p(HEMA-co-NIPAM)) cryogels using N,N-methylene-bisacrylamide (MBA), N-isopropyl acrylamide (NIPAM) and 2-hydroxyethyl methacrylate (HEMA). The study investigated the swelling capacity of p(HEMA-co-NIPAM) under different pH and temperature conditions, while also assessing its biocompatibility through a blood hemolysis test. The cryogels exhibited a microporous structure, notable swelling capacity, biocompatibility, thermal stability, and temperature-sensitive swelling characteristics [23].

The advancements in hydrogel synthesis and manipulation exemplify a concerted effort to create adsorbents with enhanced capabilities for wastewater treatment. These findings underscore the pivotal role of research in the ongoing pursuit of sustainable solutions for environmental challenges. In this study, using the exceptional properties of N-isopropylacrylamide (NIPAM) and 2-hydroxyethyl methacrylate (HEMA), a novel adsorbent material, namely poly(2-hydroxyethyl methacrylate-co-N-isopropylacrylamide) p(HEMA-co-NIPAM)) hydrogels, was synthesized. These hydrogels were tailored specifically to eliminate MB from aqueous solutions. In addition to the synthesis process, the study comprehensively investigated the adsorption isotherms and kinetics associated with this adsorbent material. By combining the favorable attributes of NIPAM and HEMA, the p(HEMA-co-NIPAM) hydrogels were prepared to exhibit enhanced adsorption capabilities, making them a promising candidate for MB removal from water. In addition to the synthesis, the study explored the fundamental adsorption behavior of the p(HEMA-co-NIPAM) hydrogels. By combining the synthesis and comprehensive analysis of adsorption characteristics, this study contributes to our understanding of the potential of p(HEMA-co-NIPAM) hydrogels as viable adsorbent materials for removing MB from aqueous solutions. The findings hold implications not only for wastewater treatment but also for the broader field of environmental remediation and sustainable water management.

## 2. Materials and methods

### 2.1. Reagents

The 2-hydroxyethyl methacrylate (HEMA, ≥ 99%), ethylene glycol dimethacrylate (EGDMA) from Sigma-Aldrich (St. Louis, MO, USA), N-isopropylacrylamide (NIPAM) from Alfa Aesar (Ward Hill, MA, USA), tetrahydrofuran (THF) from Riedel-de Haën (Seelze, Germany), MB and azobisisobutyronitrile (AIBN) from Fluka (Buchs, Switzerland) were obtained. All remaining chemicals utilized were commercially sourced and possessed the highest attainable level of purity. Water utilized in the adsorption experiments underwent purification using a Milli-Q ultrapure water purification system from Millipore (Milford, MA, USA), ensuring its quality and minimizing potential contaminants. Before utilization, the laboratory glassware was meticulously rinsed with deionized water and subjected to a dust-free environment, ensuring a pristine experimental setup. By adhering to these meticulous sourcing, preparation, and purification protocols, the study aimed to establish a robust foundation for the experimental procedures, ensuring the accuracy and reliability of the outcomes.

### 2.2. Instruments

The characterization of various aspects of the study involved the use of specialized instruments and techniques:

#### Spectrophotometric analysis

The concentration and properties of MB were assessed using an Evolution 220 UV-visible spectrophotometer manufactured by Thermo Fisher Scientific (Waltham, MA, USA).

#### pH measurement

The pH levels were determined using a digital pH-meter model PL-700PC, supplied by EZDO (Taichung, Taiwan).

#### Chemical structure analysis (FTIR)

The chemical structure of the synthesized p(HEMA-co-NIPAM) hydrogel was elucidated through Fourier-transform infrared spectrometry (FTIR) utilizing a PerkinElmer Spectrum 100 FT-IR spectrometer (PerkinElmer, Waltham, MA, USA). A universal attenuated total reflectance (ATR) sampling accessory was used to facilitate the analysis.

#### Thermal characteristic investigation (TG/DTA)

Thermal properties of the p(HEMA-co-NIPAM) hydrogel were explored using a STA 6000 thermogravimetric/differential thermal analyzer (TG/DTA) provided by PerkinElmer. The investigation encompassed a temperature range of 30–400 °C with a heating rate of 10 °C/min under a nitrogen atmosphere.

#### Surface morphology observation (SEM)

The surface morphology of the p(HEMA-co-NIPAM) hydrogel was visualized through the utilization of a ZEISS Scanning Electron Microscope (SEM) (Carl Zeiss Microscopy, Oberkochen, Germany) after coating with gold film using an acceleration voltage of 3 kV. These characterization techniques were crucial for obtaining a comprehensive understanding of the synthesized p(HEMA-co-NIPAM) hydrogel’s properties, enabling a detailed analysis of its chemical structure, thermal behavior, and surface characteristics. The use of these advanced instruments contributed to the precision and depth of the study’s findings.

### 2.3. Preparation of p(HEMA-NIPAM) hydrogels

In this study, the synthesis of p(HEMA-co-NIPAM) hydrogels was achieved through a suspension polymerization technique. The following steps were undertaken to produce the hydrogels:

Initially, 2.0 mmol of 2-hydroxyethyl methacrylate (HEMA) and 3.5 mmol of N-isopropylacrylamide (NIPAM) were combined as monomers. This mixture was dissolved in 100 mL of tetrahydrofuran (THF) and stirred for a duration of 1 h. Following the initial mixing of monomers, 40 mmol of ethylene glycol dimethacrylate (EGDMA) was introduced into 100 mL of THF. This crosslinker was then combined with the monomer solution, creating a comprehensive mixture. To facilitate the polymerization process, 100 mg of azobisisobutyronitrile (AIBN) were included in the mixture. AIBN serves as an initiator to initiate the polymerization reaction. The polymerization reaction was conducted in a controlled manner. The mixture was subjected to a temperature of 60 °C for a duration of 4 h, accomplished within a water bath setup. Following the polymerization process, the suspended hydrogels underwent a series of washing steps using distilled water. This was performed to eliminate any unreacted monomers or solvents. After thorough washing, the hydrogels were allowed to dry. The drying process occurred in an oven at 65 °C overnight.

**Figure 1:**
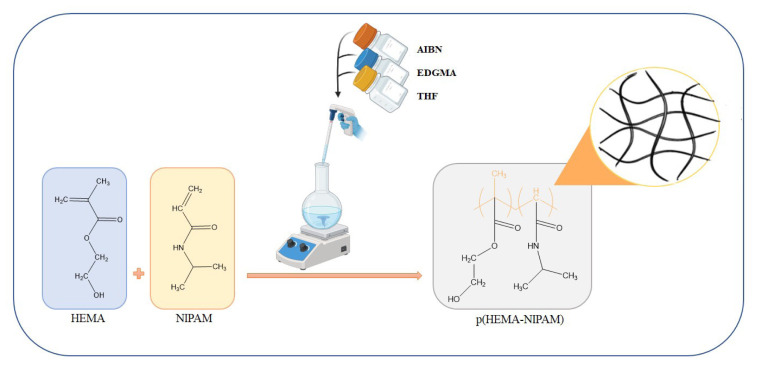
Formation scheme of p(HEMA-co-NIPAM) hydrogels.

The combination of these steps within the suspension polymerization technique resulted in the successful synthesis of p(HEMA-co-NIPAM) hydrogels (Figure 1). This method allows for the creation of hydrogels with tailored properties, enabling their potential application as an effective adsorbent material for removing MB from aqueous solutions.

### 2.4. Adsorption studies

The impact of different parameters on the adsorption capacity of hydrogels synthesized from p(HEMA-co-NIPAM) was investigated. The impact of the quantity of adsorbent on the uptake of MB by the hydrogels was analyzed over a range of polymer quantities (5.0–200.0 mg hydrogels). To evaluate the effect of contact duration, experiments were conducted for different time intervals (15–90 min). Furthermore, the connection between the initial concentration of MB and the adsorption capability of p(HEMA-co-NIPAM) hydrogels was examined within the concentration spectrum of 0.05–2.0 mg/L. Temperature variations, ranging from 15 °C to 45 °C, were employed to assess their impact on adsorption capacity. MB solutions were prepared using a universal buffer spanning pH value from 2.0 to 9.0, aiming to explore the impact of pH on MB adsorption. Subsequently, the initial and final MB concentrations in the solution were quantified using a UV-Vis spectrometer at a wavelength of 663 nm. The adsorption capacity (Q) of p(HEMA-co-NIPAM) hydrogels was calculated using the following equation:


(Eq.1) $$Q=\frac{(C_{i}-C_{f})\times V}{m},$$

where C_i_ C_f_ V and m are the initial and final MB concentrations (mg/L), volume of adsorption solution (L), and mass of the p(HEMA-co-NIPAM) hydrogels (g), respectively.

### 2.5. Desorption studies

The desorption of MB was studied on hydrogels with preadsorbed dye (initial MB concentration of 1 mg/L at 25 °C, 50 mg sorbent, pH 6.0, 1 h) in 5.0 mL of 5% hydrochloric acid (HCl) solutions at various temperatures ranging from 15 °C to 45 °C. The solution’s concentration was measured at 663 nm using a UV-Vis spectrophotometer. The percentage of dye desorption was calculated as


(Eq.2) $$Desorption\;ratio\;(\%)=\frac{\text{amount of MB desorbed}}{\text{amount of MB adsorbed}}\times 100.$$

### 2.6. Adsorption isotherms

Valuable insights into the characteristics and mechanisms of the adsorption process can be obtained by applying adsorption isotherm models such as Langmuir, Freundlich, and Temkin. To gather isotherm data, a series of MB solutions, ranging in concentrations from 0.05 to 2.0 mg/L, were brought into contact with 50 mg of the adsorbent, maintained at a consistent temperature of 25 °C. A shaking duration of 60 min was uniformly employed for all concentrations of MB to achieve complete equilibrium.

The linear expression of the Langmuir isotherm model, which assumes a monolayer adsorption phenomenon occurring on a surface with a finite count of uniformly structured adsorption sites, without transmigration of the adsorbate within the surface plane, can be expressed as [24]


(Eq.3) 1Lqm+(Ceqm),

where C_e_ is the equilibrium concentration of the MB (mg/L), q_e_ (mg/L) is the amount of dye adsorbed by the hydrogel at equilibrium and q_m_ is the monolayer adsorption capacity (mg/g) and L is related with the adsorption energy (L/mg).

An imperative dimensionless coefficient, known as the equilibrium parameter, RL, represents a fundamental aspect of the Langmuir isotherm. It was conceptualized by Weber and Chakravorti [25] and is defined as follows:


(Eq.4) $$R_{L}=1/(1+LC_{o}),$$

where L is the Langmuir constant and C_0_ is the highest initial MB concentration (mg/L).

When RL values fall between 0 and 1, they signify optimal adsorption conditions. Conversely, RL values exceeding 1 indicate unfavorable adsorption scenarios. Furthermore, RL equating to 1 denotes linear adsorption behavior, while RL of 0 signifies irreversible adsorption.

The Freundlich model is used to describe adsorption on heterogeneous surfaces or surfaces that feature sites with varying affinities. This model postulates that sites with higher binding strengths are initially occupied, with the binding strength diminishing as site occupancy increases. The Freundlich expression can be represented in a linearized form as demonstrated below [26]:


(Eq.5) $$q_{e}=\text{log }K_{f}+n_{f}\;\text{log }C_{e},$$

where K_f_ symbolizes the Freundlich constants identifying the adsorption capacity and n_f_ represents the Freundlich constants showing expediency of the adsorption process.

The Temkin adsorption isotherm elucidates the interactions between the adsorbent and adsorbate through a specific factor. According to this model, the heat of adsorption for all molecules within the adsorbed layer declines linearly. The Temkin isotherm can be defined by the following equation [27]:


(Eq.6) $$Qe=\left(\frac{RT}{bt}\right)\;\text{ln }(A\;C\text{e}).$$

In this equation, RT/b_T_ = (B (in J/mol) is the Temkin constant that is related to the heat of adsorption. A (in L/g) is the Temkin isotherm equilibrium binding constant. T (K) is the absolute solution temperature and R is the universal gas constant (8.314 J/mol K).

### 2.7. Adsorption kinetics

The equilibrium time in adsorption is determined by adsorption kinetics, and kinetic models are employed to assess both the adsorption rate (k) and the equilibrium adsorption capacity (qe). Both of these parameters play a pivotal role in understanding the adsorption mechanism. Adsorption kinetics were deduced from the data derived from the time-dependent studies. To explore the adsorption kinetics, both the Lagergren pseudo-first-order model and the pseudo-second-order model were utilized.

The pseudo-first-order model postulates that the rate-controlling step is a physisorption process involving interactions such as hydrogen bonding, van der Waals forces and π-π interactions between the adsorbate and the adsorbent. It can be defined as follows [28]:


(Eq.7) $$\text{ln}(q_{e}-q_{t})=\text{ln }q_{e}-k_{1}t,$$

where q_e_ and q_t_ are the adsorbed amounts of MB (mg/g) at equilibrium and at a given time t (min), respectively, and k_1_ is the adsorption rate constant (g/mg min). Plotting ln(qe – qt) against time (t) results in a linear graph with a slope of k1 and an intercept of ln(qe).

The pseudo-second-order model proposes that the rate-limiting step involves a chemical adsorption mechanism wherein electrons are shared or exchanged between the adsorbent and the adsorbate. The pseudo-second-order equation, which describes this process, can be presented as indicated below:


(Eq.8) $$\frac{t}{q_{t}}=\frac{1}{k_{2}q_{e}^{2}}+\frac{1}{q_{e}}t,$$

where e k_2_ (g/mg h) is the rate constant. The linear plot of t/qt versus t gives 1/q_e_ as the slope and 1/k_2_ q^2^_e_ as the intercept [29].

The adsorption procedure encompasses several stages, beginning with the diffusion of MB molecules from the aqueous phase to the surface of the adsorbent, followed by their penetration into the pores of the adsorbent. To elucidate the diffusion mechanism, the intraparticle diffusion model introduced by Weber and Morris is employed [30]:


(Eq.9) $$q_{t}=k_{pi}t^{1/2}+C_{i}.$$

The rate parameter of stage i, denoted as kpi (mg/g h1/2), can be derived from the slope of the linear relationship between qt and t1/2. The intercept of stage i, represented as Ci (mg/g), signifies the boundary layer thickness.

### 2.8. Adsorption thermodynamics

For a deeper understanding of the influence of temperature on sorption, important thermodynamic variables such as the Gibbs free energy change (ΔG°), enthalpy change (ΔH°), and entropy change (ΔS°) were determined using data acquired from temperature-dependent MB adsorption studies. The ΔG° values at different temperatures can be calculated utilizing the following equations [31]:


(Eq.10) $$\Delta G^{0}=-RTlnK_{d}$$

(Eq.11) $$K_{d}=\frac{q_{e}}{C_{e}},$$

where qe is the amount of MB adsorbed at equilibrium (in mg/g), ce is the equilibrium MB concentration (in mg/L), Kd is the distribution coefficient. ΔH° and ΔS° values are obtained from Van’t Hoff equation:


(Eq.12) $$lnK_{d}=\frac{-\Delta H^{0}}{RT}+\frac{\Delta S^{0}}{R},$$

where T is the absolute temperature and R denotes the universal gas constant (8.314 J/mol K). The slope of the equation obtained by plotting lnK_d_ values versus 1/T values gives the value of ΔH°. The intersection point of the graph gives the value of ΔS°.

## 3. Results and discussion

### 3.1. Characterization of p(HEMA-co-NIPAM) hydrogels

**Figure 2:**
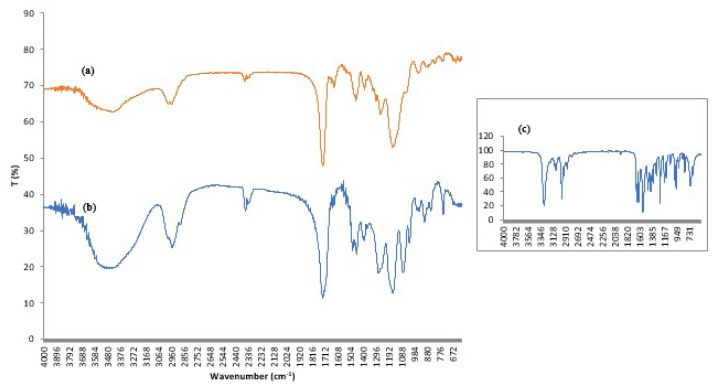
FTIR spectra of (a) p(HEMA-co-NIPAM) hydrogel (b) HEMA (c) NIPAM.

As depicted in Figure 2, the spectrum of the HEMA monomer exhibits two discernible peaks at 3429 cm^−1^ and 1720 cm^−1^, which can be attributed to the presence of hydrogen-bonded hydroxyl (–OH) groups and carbonyl (–CO) groups, respectively. The –CH stretching vibrations manifest at 2957 cm^−1^. Peaks within the range of 1075–1155 cm^−1^ correspond to –C–O–C– stretching vibrations. Figure 2 also displays the spectrum of the NIPAM monomer. The peaks at 2969 cm^−1^ indicate –CH stretching vibrations, while the peak at 1453 cm^−1^ arises from –CH bending vibrations. The observed peak at 3280 cm^−1^ is attributed to –NH stretching vibrations. The peaks at 1653 cm^−1^ and 1547 cm^−1^ result from –C=O stretching (Amide I) and –NH bending (Amide II) vibrations, respectively. The peaks belonging to isopropyl group (–CH (CH_3_)_2_) are observed at 1363 cm^−1^ and 1381 cm^−1^. Additionally, the FT-IR spectrum of the NIPAM monomer exhibits typical peaks indicating the presence of vinyl group (1410, 808 cm^−1^).

In the spectrum of the p(HEMA-co-NIPAM) hydrogel, depicted in Figure 2, the peaks ranging from 3580 cm^−1^ to 3650 cm^−1^ correspond to the vibrations of –NH and –OH groups present in both HEMA and NIPAM monomers. The –CO stretching vibrations specific to HEMA are prominent as a sharp peak at 1727 cm^−1^. Additionally, the peaks at 1387–1363 cm^−1^ can be attributed to the isopropyl group of NIPAM. The vibrations of the amide group (–CO–NH) in the structure of hydrogels are seen as peaks at 1638 cm^−1^ (–C=O stretching of Amide I) and 1543 cm^−1^ (−NH bending of Amide II). Furthermore, the spectrum of p(HEMA-co-NIPAM) also exhibits peaks at 1156 cm^−1^ and 1259 cm^−1^, attributed to the (–COO) absorption band of HEMA. Additionally, the peaks at 1048–1146 cm^−1^ in the spectrum of p(HEMA-co-NIPAM) result from C-O-C stretching vibrations. Notably, similar results have been reported in the literature for FTIR spectra of p(HEMA-co-NIPAM) hydrogels [32,33].

**Figure 3:**
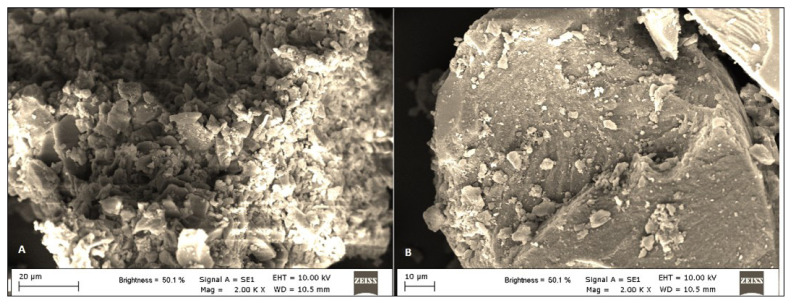
SEM micrographs of p(HEMA-co-NIPAM) hydrogel.

The SEM image of p(HEMA-co-NIPAM) gels, as depicted in Figure 3, exhibits a textured surface, indicating a highly rough structure with the potential to influence the adsorption process (Figure 3A). The discontinuous surface structure was also observed in the synthesized p(HEMA-co-NIPAM) gels (Figure 3B). The size of the p(HEMA-co-NIPAM) hydrogels was approximately determined to be 45 μm.

**Figure 4:**
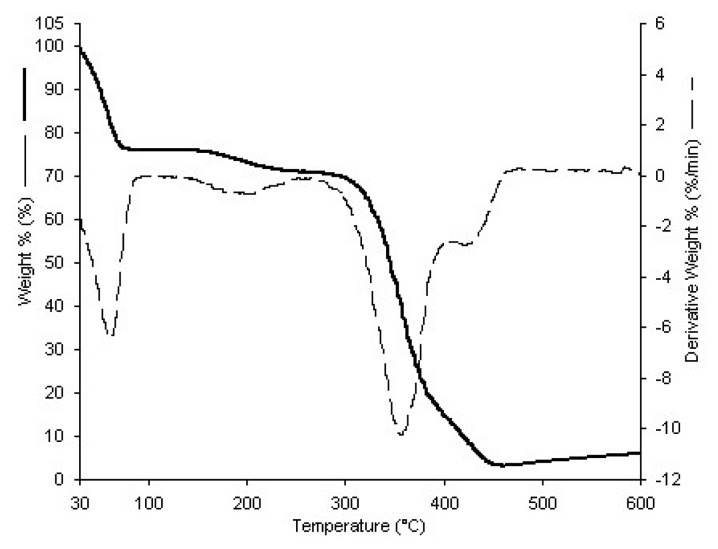
TGA curve of p(HEMA-co-NIPAM) hydrogel.

As depicted in Figure 4, the weight loss pattern for p(HEMA-co-NIPAM) hydrogel exhibits a temperature-dependent trend. The curve reveals a two-stage decomposition process. In the initial stage, occurring between 30 and 60 °C, there is an observed weight loss of approximately 18%, attributed to the evaporation of moisture content from the hydrogel. Subsequently, the second stage, deemed as the principal decomposition phase, occurs within the temperature range of 300–360 °C, resulting in a weight loss of around 67%.

### 3.2. Adsorption studies of MB

#### 3.2.1. Effect of adsorbent amount

**Figure 5:**
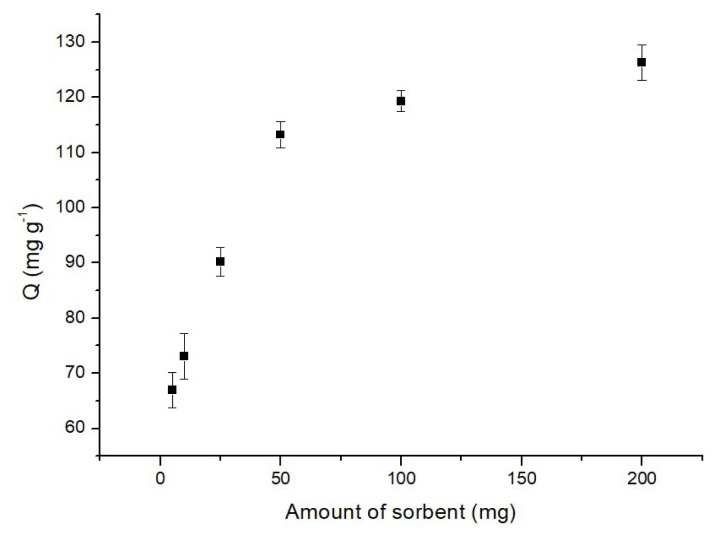
Effect of adsorbent amount on the adsorption of MB (50 mg adsorbent, 60 min, 1 mg/L MB, 25 °C, pH: 6.0).

The determination of the optimal adsorbent quantity for a given initial concentration of the analyte, along with the contact time, is a pivotal factor in adsorption practices, crucial for estimating the adsorbent’s capacity. As depicted in Figure 5, the relationship between the adsorption of MB onto p(HEMA-co-NIPAM) hydrogels and the amount of adsorbent was explored across a range of 5.0–200.0 mg hydrogels. The trends depicted in Figure 5 demonstrate that the efficiency of adsorption increases with higher amounts of adsorbent. For instance, at an adsorbent amount of 5.0 mg, the adsorption efficiency and capacity reached 50.8% and 68.0 mg/g, respectively. Subsequently, with an increase to 50.0 mg of adsorbent, the adsorption capacity peaked at 121.1 mg/g. This enhancement is predicated on the augmentation in the specific surface area and the availability of adsorption sites within the synthesized p(HEMA-co-NIPAM) hydrogels [34]. Upon further increasing the adsorbent amount to 200.0 mg, no significant changes were observed in the adsorption capacity, with the adsorption efficiency exhibiting a linear alteration. Therefore, a decision was made to select an adsorbent quantity of 50 mg for subsequent investigations, considering the optimal balance between efficiency and capacity.

#### 3.2.2. Effect of contact time

**Figure 6:**
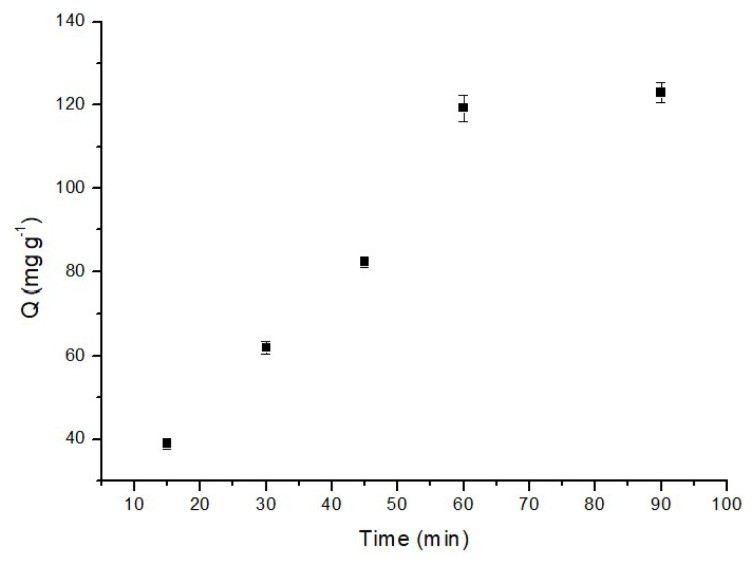
Effect of contact time on the adsorption of MB (50 mg adsorbent, 1 mg/L MB, 25 °C, pH: 6.0).

The contact time is a crucial parameter in adsorption experiments, significantly influencing the effectiveness of the adsorption process. In this study, we extensively examined the impact of contact time on the adsorption efficiency of p(HEMA-co-NIPAM) hydrogels by varying the contact time from 15 min to 90 min. As depicted in Figure 6, the adsorption capacity exhibited a value of 38.4 mg/g at a contact time of 15 min. Subsequently, as the contact time increased to 60 min, the adsorption capacity notably rose to 119.3 mg/g for MB. This observation suggests that the adsorption rate was considerably rapid within the initial 60 min, indicative of a favorable performance. After the 60-min mark, the influence of time on the adsorption rate became less significant. This trend can be explained by the sorbent reaching maximum saturation within the initial 60 min.

#### 3.2.3. Effect of initial methylene blue concentration

**Figure 7:**
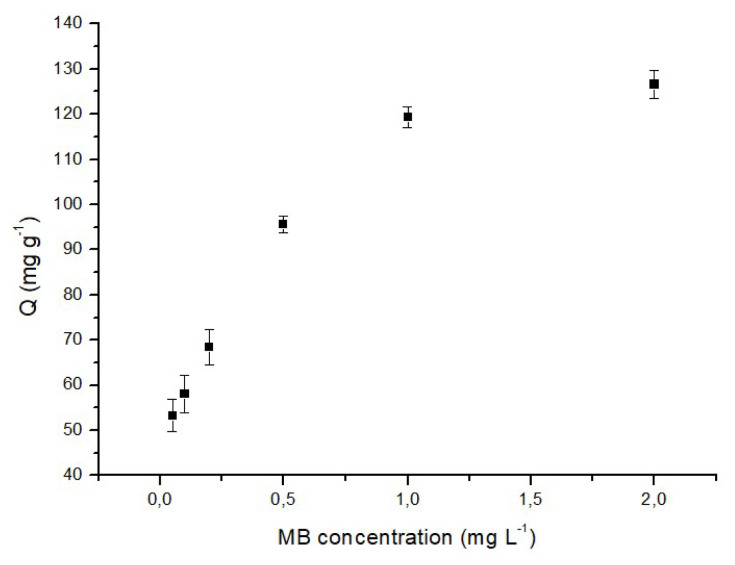
Effect of initial MB concentration on the adsorption of MB (50 mg adsorbent, 60 min, 25 °C, pH: 6.0).

Figure 7 depicts the influence of the initial concentration of MB on the adsorption efficiency onto p(HEMA-co-NIPAM) hydrogels. The data presented in Figure 7 demonstrates a significant increase in the adsorption capacity of the hydrogels, ranging from 53.5 mg/g to 119.3 mg/g, as the initial MB concentration is raised from 0.1 mg/L to 1.0 mg/L. Understanding the influence of initial dye concentration on adsorption requires consideration of the interaction dynamics between the dye molecules and the active binding regions present on the adsorbent surface. The observed enhancement in adsorption efficiency corresponding to the initial dye concentration may arise from various factors. At lower concentrations, all the accessible active sites on the adsorbent are utilized for adsorption, along with a higher rate of adsorption. As the concentration increases, the available binding sites are more effectively occupied by the dye molecules, resulting in an increased adsorption rate. However, upon reaching the point of maximum adsorption capacity, all active sites become saturated with MB molecules, leaving no additional sites for further binding. Consequently, this leads to a stabilization of the adsorption capacity percentage.

In conclusion, based on these findings, the peak adsorption capacity of p(HEMA-co-NIPAM) hydrogels was determined to be 126.6 mg/g.

#### 3.2.4. Effect of temperature

**Figure 8:**
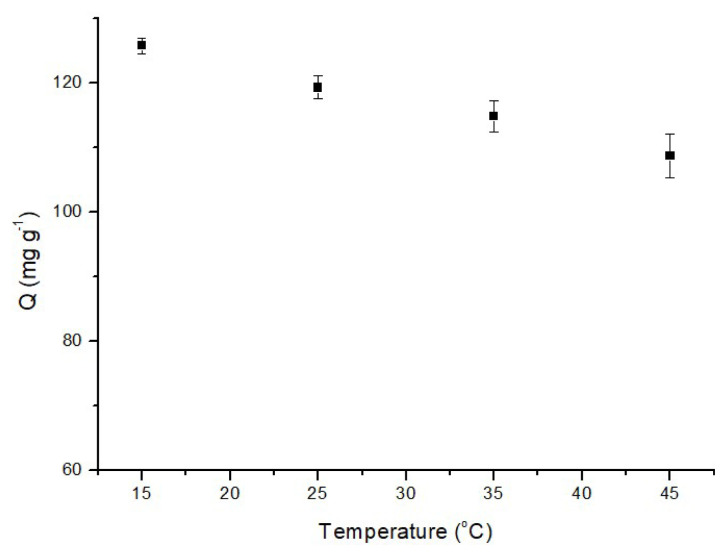
Effect of temperature on the adsorption of MB (50 mg adsorbent, 60 min, 1 mg/L MB, pH: 6.0).

The investigation of MB adsorption onto p(HEMA-co-NIPAM) hydrogels encompassed a temperature range from 15 °C to 45 °C. The results, as depicted in Figure 8, reveal a significant decrease in the adsorption capacity of the hydrogels as the temperature increases within this range. This observation aligns with the commonly recognized principle that adsorption tends to decrease as temperature rises, attributed to the exothermic nature of the adsorption process. The decrease in adsorption efficiency at higher temperatures can be attributed to changes in the interactions between the adsorbent and dye molecules. At elevated temperatures, the interaction between dye molecules and the adsorbent weakens, potentially resulting in a decrease in adsorption capacity. Concurrently, the interactions among the sorbent particles themselves may become more prominent, which could explain the observed trend.

#### 3.2.5. Effect of pH

**Figure 9:**
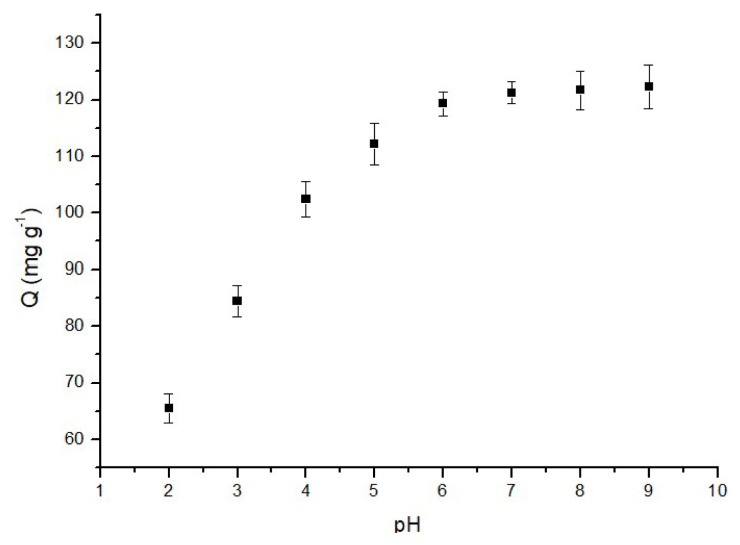
Effect of pH on the adsorption of MB (50 mg adsorbent, 60 min, 1 mg/L MB, 25 °C).

To analyze the impact of pH on the adsorption of MB onto p(HEMA-co-NIPAM) hydrogels, equilibrium batch adsorption experiments were conducted at various pH levels within the range of 2.0–9.0, all maintained at 25 °C. The results, as depicted in Figure 9, reveal an intriguing trend: the adsorption of MB onto the p(HEMA-NIPAM) hydrogel is notably less favorable under lower pH conditions. This can be attributed to the competition between the excess protons present in the solution and the cations of MB, which is particularly evident in acidic conditions.

Hydroxyl and amide NH groups are the dominant functional groups on the surface of the p(HEMA-co-NIPAM) hydrogel. Consequently, as the pH of solution increases, the density of negative charges on the sorbent’s surface also increases. This gives rise to an incremental electrostatic attraction between the hydrogel and the cationic dye molecules, ultimately leading to an enhanced adsorption rate. This results in electrostatic interactions characterized by repulsion at lower pH levels and attraction at higher pH levels.

The data depicted in Figure 9 further highlights that the adsorption capacity experiences a significant increase of approximately 46.44% as the pH rises from 2.0 to 9.0. However, the change in adsorption capacity becomes negligible above pH 6.0, with an increase of only 2.46%. Consequently, pH 6.0 was identified as the optimal pH for subsequent studies.

#### 3.2.6. Desorption studies

**Figure 10:**
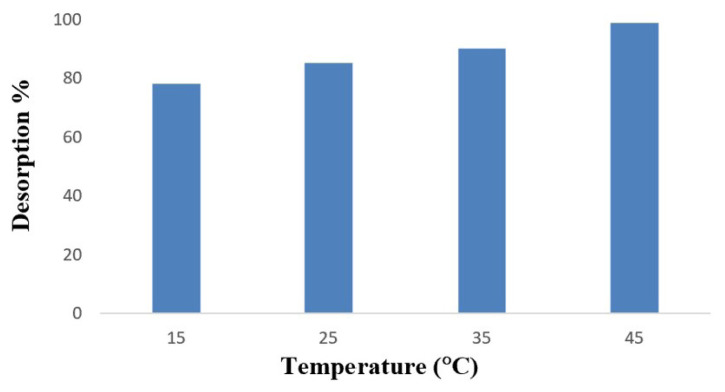
Desorption of MB adsorbed on the p(HEMA-NIPAM) hydrogel.

The desorption of MB was conducted using 5.0 mL of 5% HCl solutions at different temperatures ranging from 15 °C to 45 °C. As depicted in m Figure 10, the desorption of MB was found to be enhanced by increasing temperature.

### 3.3. Adsorption isotherms

Adsorption isotherms offer valuable insights into the adsorption mechanism, providing an understanding of the adsorption process as well as indications regarding the surface properties, affinity, and capacity of the adsorbent. The Langmuir isotherm postulates no interaction between adsorbate molecules on the isotherm binding sites, assuming a homogeneous distribution of active regions on the adsorbent surface, and is expressed by Equation (2) [23]. Conversely, the Freundlich isotherm postulates heterogeneous multilayer adsorption and is defined by Equation (4), where KF represents the Freundlich constant associated with relative adsorption capacity and the adsorbent’s affinity [26].

The summarized results of the model parameters and regression coefficients (R^2^) for the Langmuir, Freundlich, and Temkin isotherms are presented in Table 1. Notably, the R^2^ values for the Langmuir and Temkin isotherms are higher than those for the Freundlich isotherm. This indicates that the adsorption of MB onto p(HEMA-co-NIPAM) hydrogels occurred as a monolayer sorption process on a surface with homogenous sorption affinity. Additionally, the experimental qe value (126.58 mg/g) closely aligns with the calculated qm value (135.14 mg/g) obtained from the Langmuir isotherm. The calculated separation factor RL was determined to be 0.07, indicating favorable equilibrium adsorption conditions, as values between 0 and 1 are indicative of such conditions. Thus, the adsorption process on p(HEMA-co-NIPAM) hydrogels was found to be favorable in terms of affinity and capacity for the dye MB.

### 3.4. Adsorption kinetics

The dynamic experimental data were evaluated using the pseudo-first-order and pseudo-second-order kinetic models, along with the intraparticle diffusion model. The adsorption kinetics, which determine the equilibrium time, were evaluated using the MB adsorption data, and the results are presented in Table 2. The data presented in Table 2 clearly demonstrates that the correlation constant of the pseudo-second-order model is significantly greater than those of the other models. This outcome provides that the pseudo-second-order kinetic model is the most appropriate model for elucidating the adsorption of MB. Furthermore, the experimental qe and calculated qe values from the pseudo-second-order kinetic model for MB adsorption closely align, indicating that the rate-limiting step is a chemisorption or chemical adsorption process including valence forces via electron exchange or sharing between the adsorbent and the adsorbate. Based on the model, the rate constant was determined to be 5.77 × 10^−3^ g/μg h. Moreover, the utilization of the particle diffusion model unveiled a dual-phase adsorption mechanism. The initial phase suggests that MB adsorption occurs rapidly on the outer surface of p(HEMA-co-NIPAM) hydrogels due to strong electrostatic interactions. The subsequent phase signifies a slower adsorption step that reaches equilibrium. At this equilibrium state, all available adsorption sites on the hydrogels become saturated with MB molecules.

The linearity of the t/qt − t plot and the cutoff value greater than zero confirm the occurrence of intraparticle diffusion, indicating that intraparticle diffusion is not the sole rate-determining step in MB adsorption onto p(HEMA-co-NIPAM) hydrogels. Instead, it can be inferred that surface adsorption and intraparticle diffusion coexist in the process of MB adsorption.

### 3.5. Adsorption thermodynamics

Thermodynamic parameters serve as valuable tools for comprehending the impact of temperature on the adsorption process. Parameters such as changes in standard free energy (ΔG°), enthalpy (ΔH°), and entropy (ΔS°) were assessed utilizing the Van’t Hoff equation. The complete set of thermodynamic parameters is provided in Table 3.

The negative values of ΔG° provide confirmation of the spontaneous nature and possibility of the adsorption process. The observation of exothermic enthalpy changes further suggests that the adsorption process is enthalpically favorable. This notion is supported by the observed decrease in the adsorption of MB with increasing temperature.

The positive ΔS° values, particularly highlighted at 9.17 J/molK, signify heightened disorder at the solid-liquid interface throughout the adsorption procedure, indicating potential structural changes occurring in both the polymer and the MB molecules during the adsorption process.

## 4. Conclusion

In this study, p(HEMA-co-NIPAM) hydrogels were successfully synthesized using a suspension polymerization technique and employed as a support material for the removal of MB through adsorption. A comprehensive exploration was carried out to evaluate the influence of diverse adsorption parameters, encompassing sorbent quantity, initial MB concentration, temperature, pH, and contact time, on the adsorption of MB onto the p(HEMA-co-NIPAM) hydrogels.

The adsorption data in the equilibrium state exhibited a superior fit to the Langmuir adsorption isotherm, indicating that a maximum adsorption capacity of 126.58 mg/g could be achieved at 15 °C using 0.05 g of the adsorbent. Upon comparing the adsorption capacity of p(HEMA-co-NIPAM) hydrogels with reported data for other hydrogels utilized in dye adsorption (Table 4), it becomes evident that the p(HEMA-co-NIPAM) hydrogels exhibit notable competitiveness in their performance.

Kinetic data analysis revealed compatibility with both the pseudo-second-order and intraparticle diffusion models. Additionally, the application of the Van’t Hoff equation indicated that MB adsorption onto the p(HEMA-co-NIPAM) hydrogels is characterized as exothermic and spontaneous.

These findings underscore the effectiveness of the synthesized p(HEMA-co-NIPAM) hydrogels in sequestering MB dye, rendering them a promising tool for wastewater treatment, particularly in the context of eliminating pollutants from textile effluents. Consequently, there is potential for the successful adaptation of this simple, rapid, cost-effective, and environmentally friendly technique on an industrial scale.

## Figures and Tables

**Table 1:** Adsorption isotherm parameters for MB.

**Langmuir isotherm**				
*q*_e_ (mg/g)	*L* (L/μg)	*q*_m_(mg/g)	*R*_L_	*R* ^2^
126.58	6.73	135.14	0.07	0.996
**Freundlich isotherm**				
*n*_f_		*K*_f_ (mg/g)	*R* ^2^	
0.26		110.87	0.975	
**Temkin isotherm**				
A (L/mg)	*b*_T_	B	*R* ^2^	
25.08	2.21	1.12	0.996	

**Table 2:** Adsorption kinetic parameters for MB.

**Pseudo-first-order kinetic model**				
***q*** _e*,_ ** (mg/g)**	***q*** _e**_ ** (mg/g)**		***k*** *_1_* ** (1/hour)**	***R*** **^2^**
125.02	221.03		3.24	0.9651
**Pseudo-second-order kinetic model**				
***q*** _e**_ ** (mg/g)**	***k*** *_2_* ** (g/mg h)**	**t** **_½_** ** (min)**	**h** **_0,2_** ** (mg/g min)**	***R*** **^2^**
196.08	5.77 × 10^−3^	5.28	3.20	0.9859
**Particle diffusion model**				
***k*** **_p_** *_i_* ** (mg/g h** **^½^** **)**	***C****_i_* **(mg/g)**			***R*** **^2^**
90.12	8.36			0.7891

**Table 3:** Adsorption thermodynamic parameters for MB.

Δ*H*° (kJ/mol)	Δ*S*° (J/mol)	Δ*G*° (kJ/mol)			
		**288 K**	**298 K**	**308 K**	**313 K**
−9.73	9.17	−11.91	−12.46	−12.56	−12.64

**Table 4:** Comparison of adsorption capacities (mg/g) of different adsorbents.

Adsorbents	Dye	Adsorption capacity [mg/g]	Source
P(HEMA-NIPAM)	MB	126.6	This study
Ala-Car-GO hydrogel	MB	132.35	[35]
P(HEMA-*co*-DMAa) copolymer	MB	80.27	[36]
N-Isopropylacrylamide-based hydrogels	MB	22.18	[37]
SPC-SAP	MB	62.52	[38]
PANI	MB	40.2	[39]
MCFNP	MB	57.74	[40]
P(AA)/p(ES) composite hydrogel	MB	84.82	[41]
p [MAA]) hydrogel	MB	65	[42]
